# Bacterial communities and potential waterborne pathogens within the typical urban surface waters

**DOI:** 10.1038/s41598-018-31706-w

**Published:** 2018-09-06

**Authors:** Decai Jin, Xiao Kong, Bingjian Cui, Shulan Jin, Yunfeng Xie, Xingrun Wang, Ye Deng

**Affiliations:** 1 Key Laboratory of Environmental Biotechnology, Research Center for Eco-Environmental Sciences, Chinese Academy of Sciences, Beijing, 100085 China; 2 College of Resources and Environment, University of Chinese Academy of Sciences, Beijing, 100049 China; 3 School of History Geography and Tourism, Shangrao Normal University, Shangrao, 334000 China; 4 State Key Laboratory of Environmental Criteria and Risk Assessment, Chinese Research Academy of Environmental Sciences, Beijing, 100012 China

## Abstract

Waterborne pathogens have attracted a great deal of attention in the public health sector over the last several decades. However, little is known about the pathogenic microorganisms in urban water systems. In this study, the bacterial community structure of 16 typical surface waters in the city of Beijing were analyzed using Illumina MiSeq high-throughput sequencing based on 16S rRNA gene. The results showed that Bacteroidetes, Proteobacteria and Actinobacteria were the dominant groups in 16 surface water samples, and Betaproteobacteria, Alphaproteobacteria, Flavobacteriia, Sphingobacteriia and Actinobacteria were the most dominant classes. The dominant genus across all samples was *Flavobacterium*. In addition, fifteen genus level groups of potentialy pathogenic bacteria were detected within the 16 water samples, with *Pseudomonas* and *Aeromonas* the most frequently identified. Spearman correlation analysis demonstrated that richness estimators (OTUs and Chao1) were correlated with water temperature, nitrate and total nitrogen (*p* < 0.05), while ammonia-nitrogen and total nitrogen were significantly correlated with the percent of total potential pathogens (*p* ≤ 0.05). These results could provide insight into the ecological function and health risks of surface water bacterial communities during the process of urbanization.

## Introduction

Surface freshwater ecosystems including lakes, rivers and streams, play an important role in the recycling of nutrients, biogeochemical cycling, and energy flows. In these ecosystems a diverse range of microorganisms play critical roles in the functioning of these cycles, which is associated with physicochemical parameters of water^1^. In addition, the use of surface water for human recreation and consumption has increased with the pace and extent of urbanization, making microbial analysis of these water resources an essential part of water quality and human health.

A number of investigations into surface water microbial diversity have been performed^2,3^. However, the microbial communities inhabiting urban surface waters are still relatively unknown. Beijing, as one of the largest cities in China, with a population of over 20 million, contains a number of surface water ecosystems, including river, reservoir, stream and lakes, all of which are closely tied to people’s lives. A better understanding of the scale spatial characteristics of microbial population (distribution, abundance and structure, etc.). The influence of environmental factors is essential to understanding the microbial processes underlying the urban surface ecosystem and is of particular importance to water management and public health. In previous studies, Araya *et al*. studied bacterial community composition in an urban river using fluorescent *in situ* hybridization (FISH) and denaturing gradient gel electrophoresis (DGGE), and showed that stream water was dominated by *β*-Proteobacteria and the Cytophaga-Flavobacterium cluster^4^. Zhang *et al*. investigated the bacterial community structure of surface water in three rivers located in the city of Shanghai over a one-year period using DGGE, showing that temperature was the major driver of bacterial community composition^5^. Unfortunately, water microbial communities are particularly difficult to study by traditional PCR-cloning based approaches (DGGE, clone library and FISH, etc.). Recently developed high-throughput next-generation sequencing methods, such as MiSeq pyrosequencing of the 16S rRNA gene, have provided more comprehensive descriptions of bacterial communities in various environments due to the increased number of sequence reads that can be obtained. However, there is very little published research on bacterial diversity and community composition of the surface waters in Beijing. In 2015, Wei *et al*. reported the core microbial community from five surface water samples was composed of 13 phyla and 40 genera^6^. However, their correlation with physical and chemical factors in the environment remains unknown.

The objective of this study was to examine and compare the microbial diversity and abundance of sixteen surface waters in Beijing, using the Illumina MiSeq system. Meanwhile, multivariate statistical analysis was also performed to evaluate the relationships between microbial communities and environmental factors. Furthermore, the presence of potential pathogens in these water samples was also evaluated. Our results provide more detailed information on the characteristics of urban surface water microbial communities, as well as for risk management of potential bacterial pathogens in urban surface waters.

## Results

### Physicochemical characteristics of 16 surface water samples

The temperature of sampling sites ranged from 9.0 to 25.0 °C with an average of 17.65 °C. Plate counts showed the highest concentrations of total bacteria were in sample HL (3.83 × 10^6^ CFU/mL), which also exhibited the highest turbidity value, followed by sample QR and GBD, with the values of 3.0 × 10^4^ and 2.8 × 10^4^ CFU/mL, respectively, while sample MY contained the lowest number (3.0 × 10^2^ CFU/mL). The highest conductivity was found in sample QR. Total nitrogen ranged from 1.38 to 19.0 mg/L and nitrate-nitrogen from 0.8 to 17.3 mg/L. The highest of total phosphorus was observed in sample HL, and the lowest in sample CQ. The properties of 16 surface water samples are listed in Table 1.

**Table 1:** Physical and chemical properties of the 16 surface water samples.

Location	Temp (°C)	pH	EC (µS/cm)	TB (10^3^/mL)	Nitrate (mg/L)	Cl (mg/L)	NTU	AN (mg/L)	TP (mg/L)	TN (mg/L)
BJ	15.4	7.63	863	1.25	1.41	44	22	0.4	0.02	2.29
QR	19.6	7.76	1315	30	16	139	38	0.75	0.1	17.7
ZGC	17	7.66	742	1.15	1.53	38	44	0.07	<0.01	2.5
WY	19	6.17	914	13.1	1.78	79.2	<3	0.26	0.03	2.69
RM	19	6.8	848	2.75	1.09	80.1	21	0.82	0.04	2.44
HR	19	6.79	401	0.5	0.48	36	10	0.10	0.04	1.28
HL	18	6.89	328	3835	0.71	18.1	59	2.07	0.69	4.3
YX	15	6.57	331	0.55	1.48	11	<3	0.1	0.03	2.5
QL	16	6.92	289	9.5	7.46	9.05	7	0.55	0.21	8.86
MY	9	6.45	330	0.3	0.8	13.5	<3	0.09	0.03	1.38
YD	17	8.28	955	3.1	1.61	165	19	0.24	0.15	2.69
GBD	25	7.58	848	28.4	17.3	103	30	0.83	0.32	19
CQ	18	7.87	472	2.2	1.24	26.8	9	0.07	<0.01	1.89
NT	18.5	8.84	1011	4.55	3.14	161	18	0.35	0.18	4.89
NH	19	7.83	733	3	17.4	104	7	0.28	0.04	18.3
QH	18	8.4	546	0.7	3.56	43.8	<3	0.11	0.05	3.91

Temp: Temperature. TB: Total Bacteria. Chloride: Cl. NTU: Turbidity. AN: Ammonia nitrogen. TP: Total phosphorus. TN: Total nitrogen.

### Diversity analysis using operational taxonomic units

In total, Illumina MiSeq sequencing generated clean reads of 585,728 high quality 16S rRNA gene sequences with an average of 36,608 sequences per sample. After trimming, alignment, and chimera removal using the standard operating procedure for Mothur, the number of sequences of each sample ranged from 17,519 (QR) to 36,002 (WY). All samples were randomly resampled to 17,519 sequences, with 280,304 rarefied sequences for the 16 samples retained and were subjected to further statistical analyses.

Biodiversity of the 16 surface water samples was investigated by analyzing OTUs, Chao1, and Shannon indices at cut-off levels of 3% (Table 2). Slight differences in biodiversity were observed among the different sites. For example, the library derived from sampling site CQ had the greatest number of OTUs (1,483), followed by sample GBD (1,424) and QR (1,406), while sample ZGC had the smallest (393). Chao1 numbers were considerably higher than OTU numbers, suggesting that more OTUs may exist in these bacterial communities. However, the Good’s coverage value of all samples was above 95.14%, suggesting that the observed sequences could function as a good representation of the bacterial communities present in the 16 samples. Additionally, the Shannon diversity index was also generated for each sample, with the results supporting that sample CQ had the highest bacterial diversity of the 16 samples.

**Table 2:** Diversity indices from 16 surface water samples.

Sample	Resample reads	OTUs	Chao1	Shannon	Simpson	Coverage (%)
BJ	17519	794	2170	4.14	0.03	97
CQ	17519	1483	3260	5.06	0.02	95
GBD	17519	1424	3338	4.99	0.02	95
HL	17519	1063	2728	4.57	0.03	96
HR	17519	731	1756	4.30	0.03	97
MY	17519	709	1581	4.52	0.02	98
NH	17519	1332	3291	4.61	0.04	95
NT	17519	936	2622	4.36	0.03	96
QH	17519	826	2616	4.12	0.03	97
QL	17519	657	1790	3.63	0.06	97
QR	17519	1406	3622	4.48	0.04	95
RM	17519	906	2398	4.25	0.03	96
WY	17519	882	2232	4.21	0.05	97
YD	17519	1077	2912	4.70	0.02	96
YX	17519	773	1738	4.39	0.03	97
ZGC	17519	393	1158	2.70	0.15	98

The OTU, Chao1, Coverage, Shannon and Simpson parameters are presented for a dissimilarity of 3.

In order to test the interaction between biodiversity indices and water quality indices, Spearman’s rank correlation was calculated to compare the correlation between diversity indices (OTUs, Chao1 and Shannon diversity index) and water temperature as well as other qualitative parameters. As indicated by Spearman’s rank correlation, water temperature, nitrate, and TN had significant correlations (*p* < 0.05) with OTUs and Chao1 (Table 3). Among all the water quality indices, only the TN was significantly positively correlated with Chao1 at *p* ≤ 0.01 level. It should be noted that no significant correlation was found between Shannon index and any water physicochemical parameter.

**Table 3:** Correlation between the alpha diversity and water quality indexes.

	Richness	Chao1	Shannon
Temperature	0.55^*^	0.60^*^	0.23
pH	0.32	0.49	0.08
EC	0.36	0.50	0.06
TB	0.09	0.11	0.13
Nitrate	0.61^*^	0.62^*^	0.25
Chloride	0.46	0.60^*^	0.28
Turbidity	0.13	0.19	−0.16
AN	0.28	0.32	0.19
TP	0.23	0.27	0.23
TN	0.62^*^	0.64^**^	0.26

Significant correlation coefficient at: *p ≤ 0.05; **p ≤ 0.01.

### Taxonomic distributions among samples

**Figure 1:**
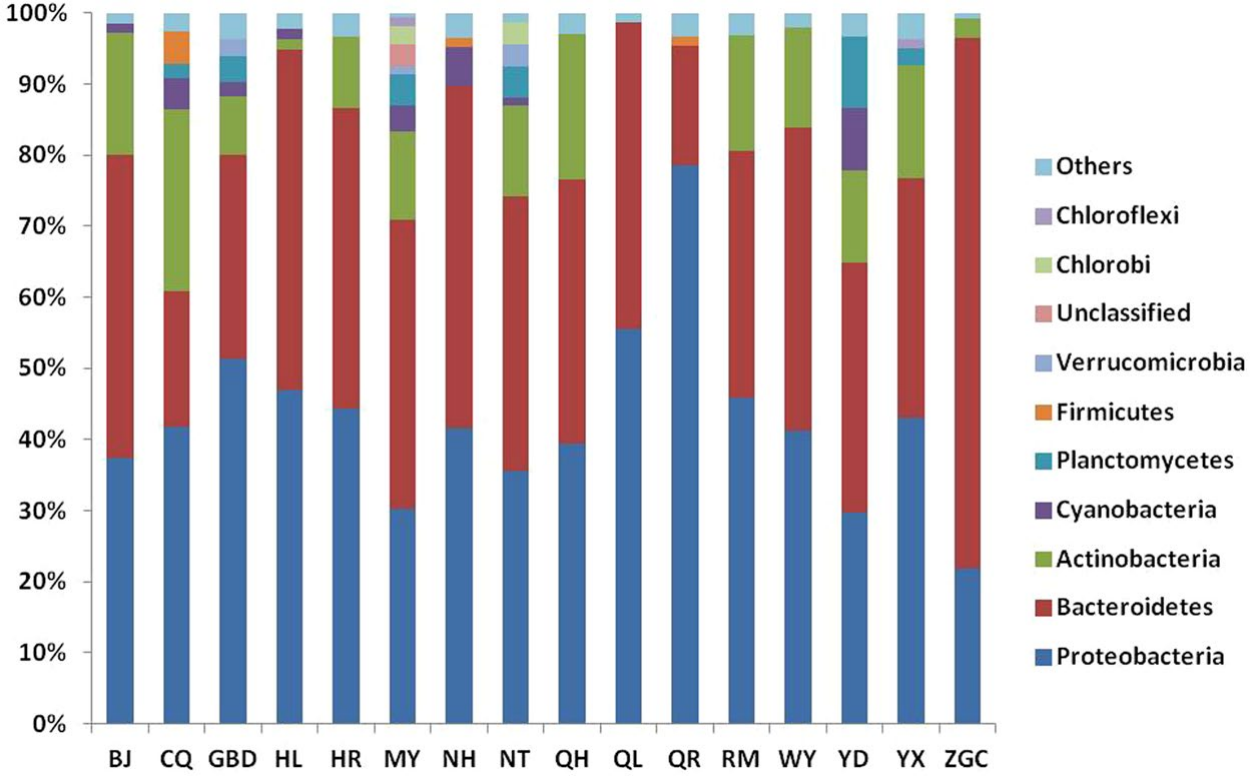
Bacterial community compositions at the phylum level as revealed by MiSeq sequencing. Phyla making up less than 1% of total composition were classified as “others” in the 16 samples.

Surface water OTUs were classified to 43 bacterial phyla the most predominant phyla being Proteobacteria, Bacteroidetes and Actinobacteria. Based on relative abundances, the dominant groups (greater than 1% abundance) of each sample are displayed in Fig. 1. As shown, Proteobacteria and Bacteroidetes were the most commonly detected phyla in all the samples, accounting for 21.9–78.5% and 19.1–74.7% of sequences, respectively. The sample QR contained the most Proteobacteria (78.5%), while sample ZGC contained the most Bacteroidetes (74.7%). At the class level, the dominant groups for the 16 water samples varied. For instance, Betaproteobacteria were dominant in the majority of water samples including BJ, GBD, HR, NT, QH, QL, QR, RM, WY and YX, while in samples HL, NH, YD and ZGC, Flavobacteriia were the dominant group. It should be noted that only sample CQ was dominated by the Actinobacteria class, and Sphingobacteriia was dominant in only sample MY. Of the 16 samples, only in MY were there OTUs (2.97%) that could not be assigned to any known, which may indicate a novel uncharacterized phylum present at that site.

**Figure 2:**
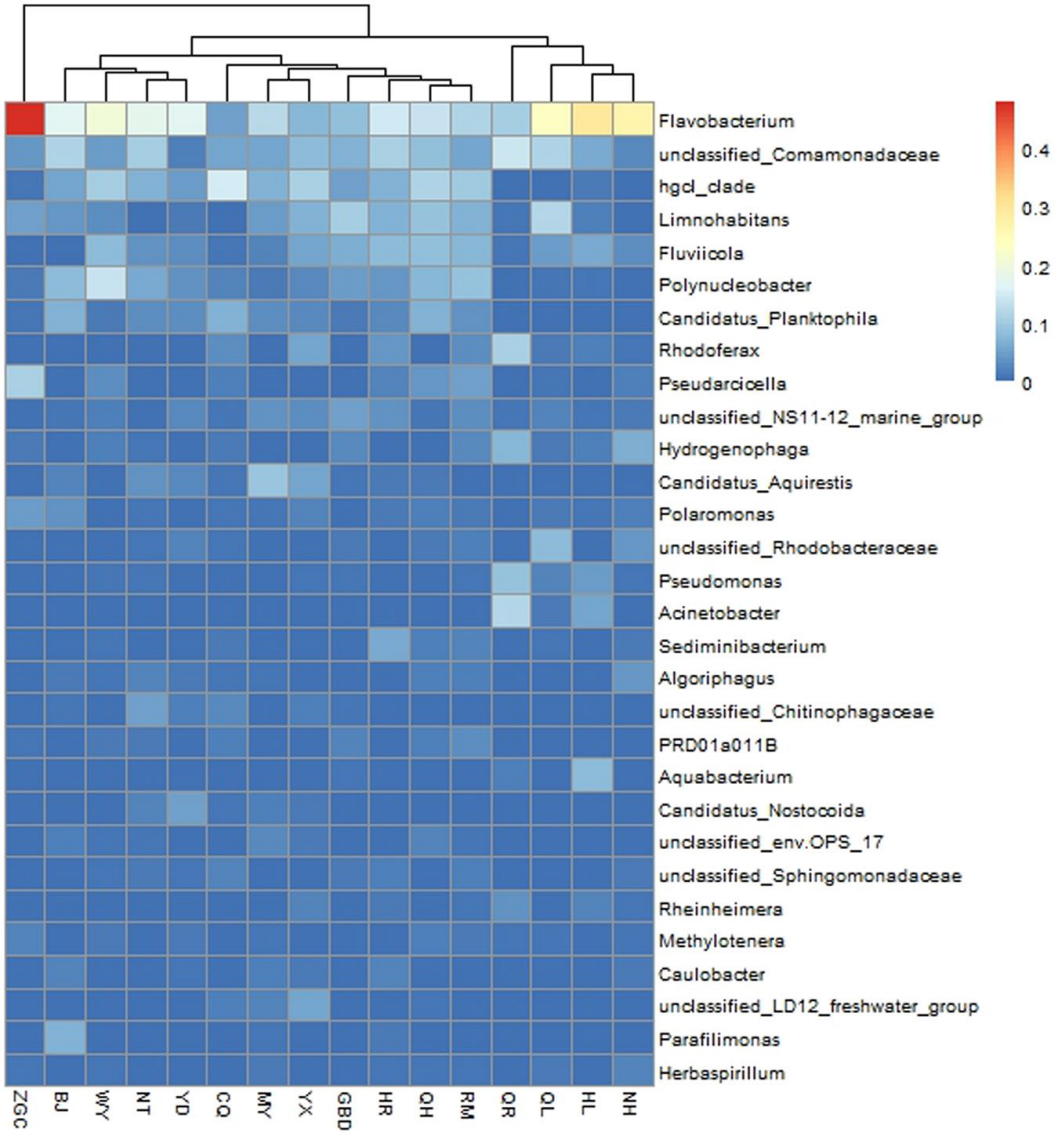
Heat map of the top 30 genera present in the sixteen surface water samples.

The abundance of the top 30 genera are shown in Fig. 2. The most predominant genera among Proteobacterial sequences were *unclassified_Comamonadaceae, Limnohabitans, Polynucleobacter, Rhodoferax, Hydrogenophaga, Candidatus_Aquirestis* and *Polaromonas. Flavobacterium* was the predominant Bacteroidetes genus and was the most predominant genus in 12 of 16 surface water samples. The exceptions being CQ, GBD, QR and WY.

**Figure 3:**
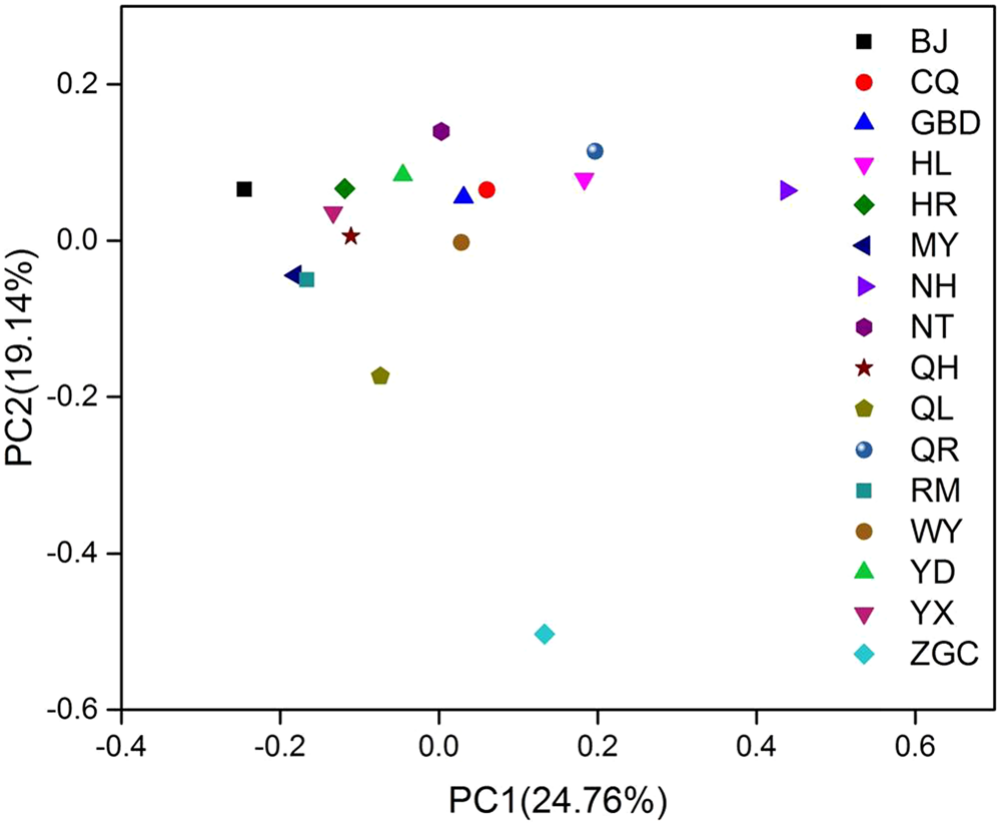
The plots of principal coordinate analysis illustrating community dissimilarities based on weighted UniFrac distances metric.

Principal coordinate analysis was used to analyze the bacterial community dissimilarity. As shown in Fig. 3, significant differences in bacterial community composition were found among most samples. However, similar patterns of bacterial community composition were observed for few samples, as exemplified by samples MY and RM.

**Figure 4:**
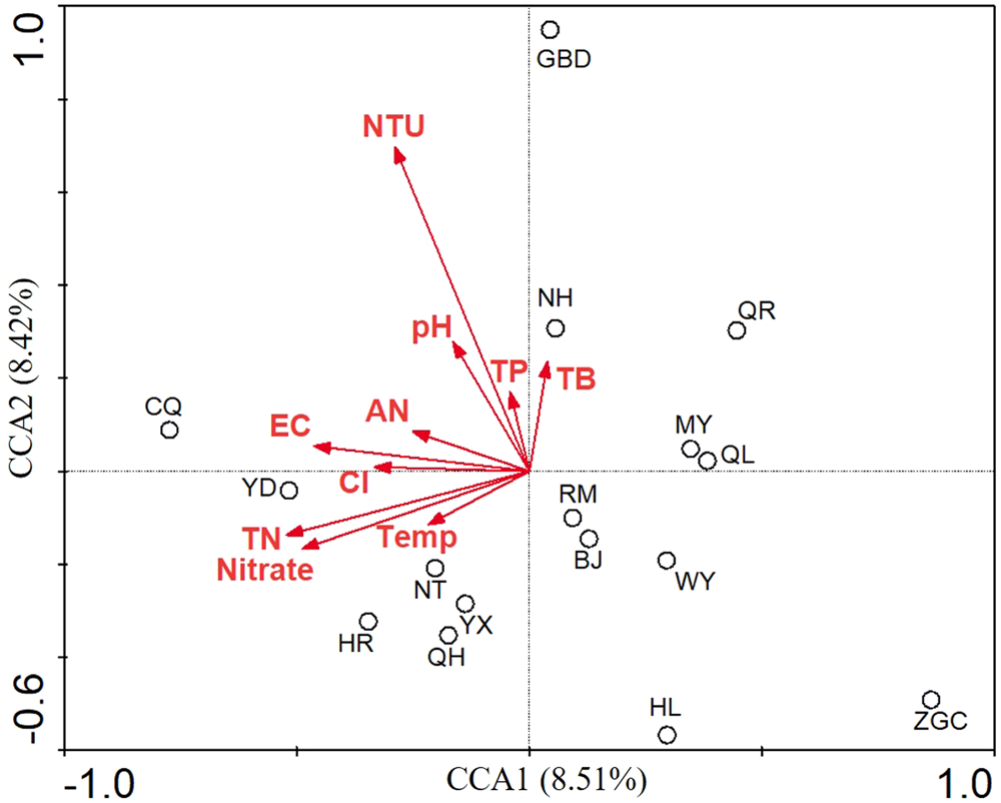
Canonical correspondence analysis (CCA) biplot of bacterial communities and environmental parameters.

To test possible linkages between the microbial communities and surface water quality parameters, canonical correspondence analysis (CCA) was performed (Fig. 4) and the results showed that bacterial community composition was significantly correlated to turbidity (*r*^2^ = 0.56; *p* = 0.01). CCA axis 1 was positively correlated with water temperature, pH, EC, nitrate, chloride, turbidity, AN, TP and TN, but was negatively correlated with TB. The first CCA dimension explained 8.51% of the variation of bacterial communities, and the second explained 8.42%.

### Detection of potential waterborne pathogens

**Figure 5:**
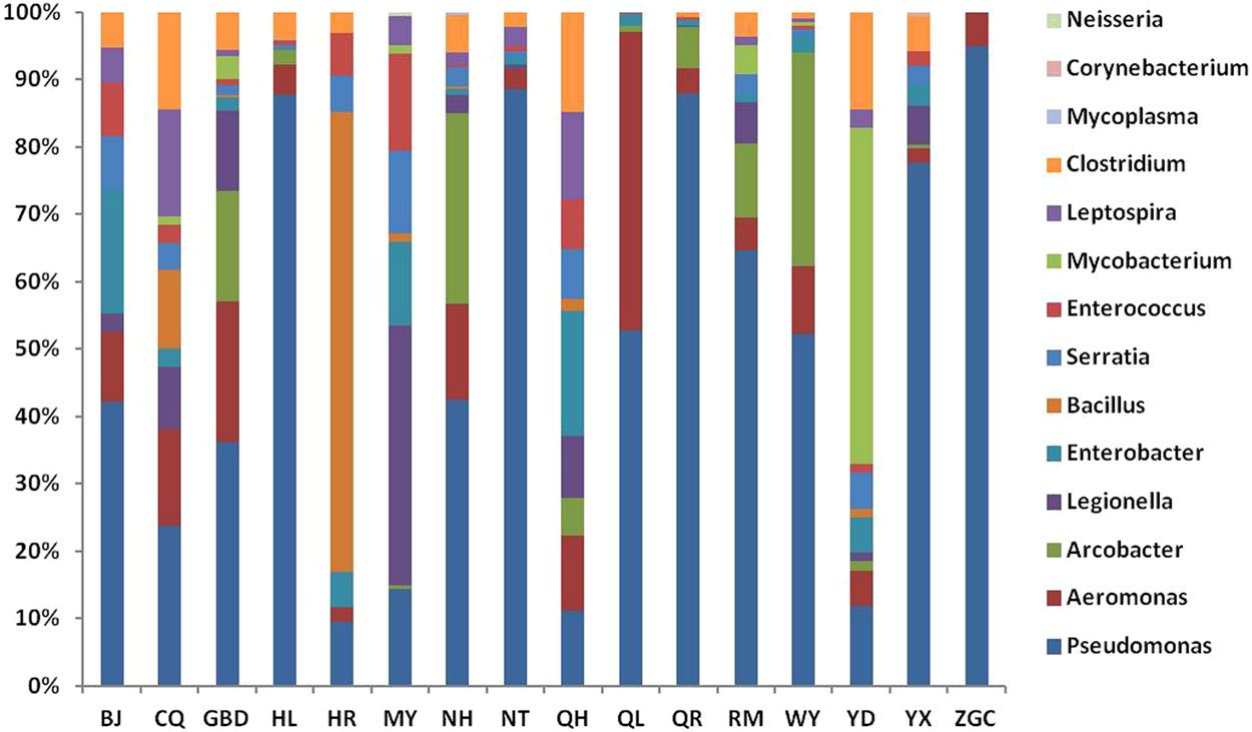
Potential pathogenic bacteria diversity and abundance at genus level in total pathogen.

Of the 66 potential waterborne pathogens recommend by USA EPA^7^, a total of 15 were detected across all of the samples. Only two types of potential pathogenic bacteria were found in sample ZGC, while 12 were detected in sample NH, the most of any sample. However, the greatest abundance of potential waterborne pathogens was observed in sample QR, accounting for 10.39% of all the OTUs, followed by sample HL (5.33%), QL (3.85%), GBD (1.94%), NH (1.26%), WY (1.34%), YX (1.07%), MY (0.96%), RM (0.94%), NT (0.80%), HR (0.56%), CQ (0.43%), YD (0.43%), QH (0.31%), BJ (0.23%) and ZGC (0.11%). At the phylum level, Proteobacteria contributed the highest proportion of bacterial richness that may be caused diseases in humans, accounting for 24.48–100% in all the potential pathogenic bacteria composition. The next most dominant phylum was the Firmicutes, followed by Actinobacteria, Spirochaetae and Tenericutes. The distribution of potential pathogenic bacteria at genus level is shown in Fig. 5.

Among the potential bacterial pathogens, the genus *Pseudomonas* was dominant in all the samples expect for sample YD, which was dominated by the genus *Mycobacterium*. The next most dominant flora in each sample was different. For instance, *Aeromonas* was the second richest genus of potential pathogenic bacteria in samples CQ, GBD, HL, NT, QH, QL and YX. While in samples NH, QR, RM and WY, *Arcobacter* was the second most abundant genus of potential pathogenic bacteria among all the OTUs. Additionally, the relationship between water parameters and the percent of total potential pathogens was assessed using Spearman correlation coefficient analysis (Table 4). The results indicated that both AN and TN were significantly correlated with the percent of total potential pathogens (*p* ≤ 0.05). No significant links were found between the percent of total potential pathogens and the remaining water parameters.

**Table 4:** The Spearman correlation coefficients between total pathogen percent (%) and water quality indexes.

	Total Percent (%)
Temperature	0.50
pH	0.81
EC	0.30
TB	0.18
Nitrate	0.05
Chloride	0.53
Turbidity	0.06
AN	0.02^*^
TP	0.10
TN	0.03^*^

Significant correlation coefficient at: *p ≤ 0.05; **p ≤ 0.01.

## Discussion

Surface water, which consists of both natural and man-made water bodies (rivers, lakes, reservoirs, wetlands, parks, etc.) plays a vital role in urban ecosystem services^8,9^, and remains an important source for drinking water, irrigation, and recreation, etc. Changes in urban surface water may result in fluctuation of microbial community, with consequences to water quality and water-borne pathogen contamination. Assessing and understanding the microbial ecology of urban surface water from different regions is essential for making water quality management plans. In our study, high-throughput amplicon sequencing was used to characterize bacterial community structure and diversity of 16 surface water samples collected from various locations in the city of Beijing. The total number of phyla in each of the 16 samples ranged from 9 to 37, while 20 to 27 phyla have been observed in five surface water samples collected from other areas of Beijing^6^. These results suggest that the bacterial community composition of Beijing surface waters was significantly different from each other. The dominant phyla also differed among the 16 surface water samples. Proteobacteria (21–53%) and Bacteroidetes (11–64%) were the dominant phyla, which is in agreement with the findings of Bai *et al*.^10^, who examined the bacterial community structures in wastewater treatment bioreactors via high-throughput sequencing. However, Actinobacteria (41.17% of total sequences) and Proteobacteria (31.80%) were the dominant phyla in the Ganjiang River basin^11^, similar results have been found in other Beijing urban surface water bodies^6^.

In previous studies, the factors that could influence the bacterial community in surface water were well understood. For example, the bacterial community composition in the Danube River was determined by denaturing gradient gel electrophoresis analysis, and results indicated its shifts were related to changes in environmental conditions^12^. In 2015, Zhong *et al*. reported that the composition and diversity of the prokaryotic communities were significantly related to the concentrations of salinity in lakes of the Tibetan Plateau^13^. Recently, Ibekwe *et al*. reported that microbial community structure of an urban river was significantly shaped by several key physical and chemical variables for water, including NO_2_, pH, and NO_3_^14^. Temperature was found to be the most influential factor in determining bacterial community composition of Ganjiang River^11^. In contrast to these studies, we found turbidity significantly affected bacterial community. This may be because the 16 water samples showed a slightly different turbidity.

In many countries, especially in the developing world, surface waters are severely contaminated with pathogens leading to various waterborne disease outbreaks^15^. However, few studies concerning waterborne pathogens in urban surface waters have utilized high throughput sequencing. In the present study, it was found that potentially pathogenic bacteria were ubiquitous across all of the sampled surface waters. Among the potential waterborne pathogens, *Pseudomonas* spp. were the most prevalent at all sites; followed by *Aeromonas*. Several other potentially pathogenic genera, such as *Arcobacter*, *Legionella*, *Enterobacter*, *Bacillus*, *Serratia*, *Enterococcus*, *Mycobacterium*, *Leptospira*, *Stenotrophomonas*, *Clostridium*, *Mycoplasma*, *Corynebacterium* and *Neisseria* were also detected in various samples. Examples of how potentially pathogenic these organisms include the following examples: Between 2007 and 2009, 21% of the waterborne outbreaks caused by bacteria was reported in the United States, the leading etiologic pathogen bacteria including *E. coli* O157: H7, *Shigella sonnei*, *Pseudomona*s spp., and *Legionella* spp^16^. Among species of *Legionella*, a majority are considered pathogenic, with *L. pneumophila* the leading cause of severe pneumonia worldwide^17^. Members of the *Aeromonas* genus are common aquatic microorganisms and a number of them have been associated with human diseases^18^. Several species of *Mycobacteria* are also pathogenic to humans or animals, causing pulmonary and cutaneous disease, lymphadenitis, and disseminated infections, especially in the growing immunodeficient population^19^. In addition, three waterborne outbreaks associated with *Arcobacter* have been previously reported^20^.

It should be noted that both AN and TN correlated strongly with the percent of total potential pathogens (*p* ≤ 0.05), and potentially pathogenic bacteria were present at relatively high concentrations in samples QR (10.39%), HL (5.33%) and QL (3.85%). As the short reads generated by Illumina MiSeq platform cannot be accurately classified to the species level nor can they provide information on the amount of total or viable cells in the water samples. This limitation can lead to overestimation of the abundance of potential pathogens. Even so, more attention should be paid to health risks from surface waters in urban areas.

## Conclusion

In conclusion, microbial community characteristics of 16 surface water samples in the Beijing area were studied using 16S rRNA gene amplicon sequencing. Bacteroidetes, Proteobacteria and Actinobacteria were the predominant phyla across all the samples. Principal coordinate analysis indicated significant differences in surface water bacterial community composition. Meanwhile, our results demonstrated the presence of potential waterborne pathogens in all of the surface waters sampled and that *Pseudomonas* spp. are ubiquitous bacteria in a wide variety of surface waters. Spearman correlation analysis showed a positive correlation between water quality properties (AN and TN) and total percent of the potential bacterial pathogens. These results give a better understanding of the urban surface waters ecology.

## Materials and Methods

### Study areas and sample collection

**Figure 6:**
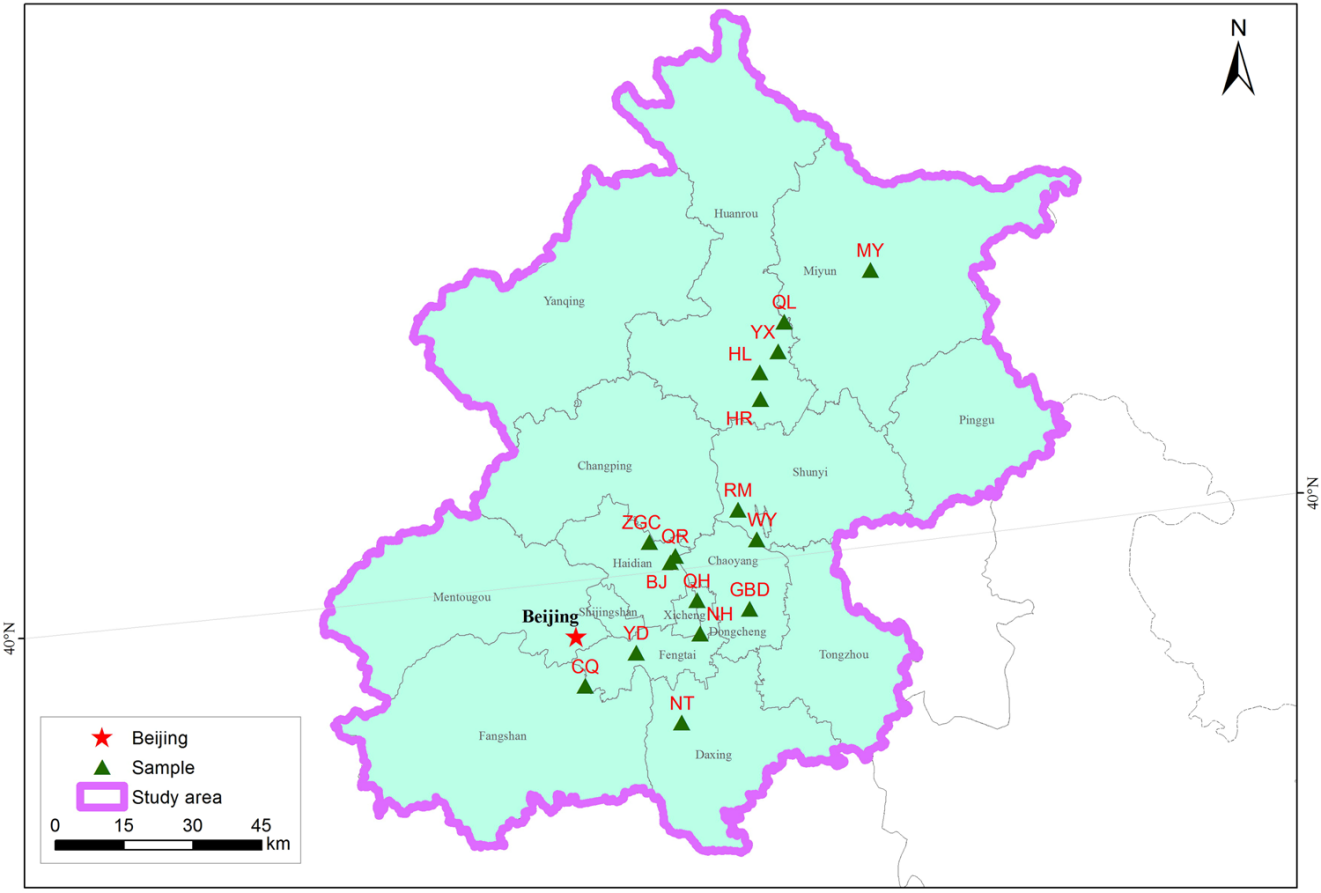
Map of sampling site locations in the Beijing city. The map was generated using Esri ArcGIS v10.1 (Redlands, CA).

From March 26^th^ to April 17^th^ in 2014, samples from the 0–15 cm depth were taken from the lakes, rivers, or reservoir banks using ethanol-disinfected core tubes and stored in sterile Nalgene sampling bottles at 4 °C until processed within 24 h. These 16 surface waters are distributed across Beijing and consequently are located in different districts (Fig. 6), i.e., Bajia country park (BJ), Qing river (QR), Zhongguancun forest park (ZGC), Wenyu river (WY), Rome lake (RM), Huairou reservoir (HR), Hongluo lake (HL), Yanxi lake (YX), Qinglong lake (QL), Miyun reservoir (MY), Yongding river (YD), Gaobeidian reservoir (GBD), Chongqing reservoir (CQ), Niantan reservoir (NT), Nanhucheng river (NH) and Qianhai park (QH). All samples were collected in triplicate and then combined to create a composite sample. The temperature (Temp), electrical conductivity (EC) and pH of the samples were determined on-site using a Multi-parameter water quality meter (YSI, USA). Other analyses included assessment of turbidity, chloride, nitrate, ammonia nitrogen, total phosphorus, and total nitrogen were performed according to Chinese national standards (GB: 5749-2006) by Beijing ZKLH Analyzing & Testing Center (Beijing, China). For determination of total bacterial count, microbial biomass was harvested from approximately volume water samples using 0.45 µm polycarbonate membranes by means of a hand-operated pump, and then placed on aerobic count chromogenic plate (Qingdao Hope Bio-Technology Co., Ltd., Shandong, China). Typical colonies were counted and results expressed as colony forming units (CFU) ml^−1^ after incubated at 37 °C for 48 h. The experiment was repeated in triplicate.

### DNA extraction from water samples and sequencing

Total bacterial DNA was extracted from suitable volumes of water samples from each site using Power Soil and Water DNA kits (MO BIO, Inc., Solana Beach, CA), according to the manufacturer’s protocol. The quality and quantity of the isolated nucleic acids were determined using the Nanodrop ND1000 (NanoDrop Technologies, Delaware, USA) and adjusted to 20–30 ng/µL with sterilized ultra-pure water. The V4-V5 regions of the bacterial 16S rRNA gene were amplified by PCR using primers 515F (5′-GTGCCAGCMGCCGCGG-3′) and 907R (5′- CCGTCAATTCMTTTRAGTTT-3′). PCR reactions were performed using 50 μL mixtures containing 5 μL of 10× PCR Buffer, 4 μL of 2.5 mM dNTPs, 1.5 μL of each primer (5 μM), 0.5 μL of *Taq* DNA polymerase, and 1 μL of template DNA, and sterilized ultrapure water to bring volume up to 50 μL. A negative control containing all reagents except the template DNA was included with each set of reaction mixtures. PCR conditions were as follows: initial denaturation at 94 °C for 5 min; followed by 35 cycles of 94 °C for 30 s, annealing at 52 °C for 30 s, and extension at 72 °C for 1 min; and a final extension at 72 °C for 10 min. PCR products were extracted from 1% agarose gels and purified using the AxyPrep DNA Gel Extraction Kit (Axygen Biosciences, USA) according to the manufacturer’s instructions and quantified using QuantiFluor^TM^-ST (Promega, USA). All PCR products were sequenced using the Illumina MiSeq platform by the Shanghai Majorbio Bio-pharmTechnology Co., Ltd., China.

### Data analysis

Sequences obtained from the Illumina sequencing platform were strictly filtered to obtained clean data and each sample was split by Qiime (V1.7.0, http://qiime.org/index.html). Low quality sequences were identified by denoising and filtered out of the dataset. The data generated by Illumina MiSeq was analyzed using the quantitative insights into microbial ecology (QIIME 1.7) toolkit^21^. Chimeric sequences were removed using the Uchime function with the sequence collection as its own database in the Mothur program. The effective sequences were then clustered into operational taxonomic units (OTU) at 97% sequence similarity with standard UCLUST method^22^. Finally, the RDP classifier was used to assign representative sequence to the microbial taxa^23^. Phylogenetic trees were then built from all representative sequences using the FASTTREE algorithm^24^. Heatmaps were drawn using gplots^25^ in R. The differences in overall community composition between each pair of samples were determined using the weighted and unweighted UNIFRAC metric^26^. For the statistical analysis, we used a randomly selected subset of 17,519 sequences per water sample to compare relative differences between samples. Normalized libraries were used to calculate the rarefaction curves and phylogenetic diversity. Rarefaction curves are a result of repeated randomizations of the observed species accumulation curve, allowing the observed richness to be compared among samples^27^. The Good’s coverage estimator was used to estimate the percent of the total species represented in a sample. Canonical correspondence analysis (CCA) was used to evaluate the relationship between the bacterial diversity revealed by pyrosequencing of 16S rRNA genes and water properties. The data of total bacteria was standardized using ln (CFU/mL) transformed before the analysis. CCA were performed using R (2.14.0, http://www.r-project.org/) with the community ecology package “vegan (2.0-4)”^28^. Spearman correlation was applied to identify the associations between water parameters and the percent of total potential pathogens, *p*-values < 0.05 were considered significant.

### Nucleotide sequence accession numbers

The sequences obtained in this study are available in the NCBI Nucleotide Archive database under project identification numbers SRR5405060-62, SRR5405064-65, SRR5405069-70, SRR5405072-75, SRR5420965-68 and SRR5429450.
